# The generation of consensus guidelines for carrying out process evaluations in rehabilitation research

**DOI:** 10.1186/s12874-018-0647-y

**Published:** 2018-12-29

**Authors:** P. Masterson-Algar, C. R. Burton, J. Rycroft-Malone

**Affiliations:** Bangor Institute for Health & Medical Research, School of Healthcare Sciences, Bangor University, Ffriddoedd Road, Bangor, UK

**Keywords:** Consensus guidelines, Process evaluation, Nominal group technique, Rehabilitation research, Complex interventions

## Abstract

**Background:**

Although in recent years there has been a strong increase in published research on theories (e.g. realist evaluation, normalization process theory) driving and guiding process evaluations of complex interventions, there is limited guidance to help rehabilitation researchers design and carry out process evaluations. This can lead to the risk of process evaluations being unsystematic. This paper reports on the development of new consensus guidelines that address the specific challenges of conducting process evaluations alongside clinical trials of rehabilitation interventions.

**Methods:**

A formal consensus process was carried out based on a modified nominal group technique, which comprised two phases. Phase I was informed by the findings of a systematic review, and included a nominal group meeting with an expert panel of participants to rate and discuss the proposed statements. Phase II was an in depth semi-structured telephone interviews with expert panel participants in order to further discuss the structure and contents of the revised guidelines. Frequency of rating responses to each statement was calculated and thematic analysis was carried out on all qualitative data.

**Results:**

The guidelines for carrying out process evaluations within complex intervention rehabilitation research were produced by combining findings from Phase I and Phase II. The consensus guidelines include recommendations that are grouped in seven sections. These sections are theoretical work, design and methods, context, recruitment and retention, intervention staff, delivery of the intervention and results. These sections represent different aspects or stages of the evaluation process.

**Conclusion:**

The consensus guidelines here presented can play a role at assisting rehabilitation researchers at the time of designing and conducting process evaluations alongside trials of complex interventions. The guidelines break new ground in terms of concepts and theory and works towards a consensus in regards to how rehabilitation researchers should go about carrying out process evaluations and how this evaluation should be linked into the proposed trials. These guidelines may be used, adapted and tested by rehabilitation researchers depending on the research stage or study design (e.g. feasibility trial, pilot trial, etc.).

**Electronic supplementary material:**

The online version of this article (10.1186/s12874-018-0647-y) contains supplementary material, which is available to authorized users.

## Background

- Rehabilitation interventions are often complex, hence, their investigation can be particularly demanding [1, 2]. Complex interventions can be defined as those made up of a number of components or *active ingredients* that interact with each other and with outside factors to bring about changes to outcomes [3]. Complex interventions are regarded as having inherent heterogeneity [4]. They will often be offered multiple times to multiple participants, the location and site of delivery can change as well and they can be delivered to individuals, families, combinations, etc. [5]. Similarly, they are designed in a number of sessions to allow time for individuals to learn and comprehend their content [6]. Rehabilitation interventions are complex and present a number of specific challenges: They often involve complex behavioural treatments in contrast to passive or surgical treatments [7].
- They are often delivered face to face, where personal interactions and relationships play an important role in influencing patient engagement.
- They are linked treatment plans which will need to be tailored to patients’ needs, and wider social circumstances.
- They are context specific and defined as the interaction between the individual and the environment [8]. In other words, rehabilitation interventions can be shaped by the wider environmental and therapeutic milieu in which it is practiced.

Because of these particular characteristics, it can be extremely difficult to know why rehabilitation interventions work (or not). Hence, rehabilitation research is highly challenging for a number of reasons. Firstly, rehabilitation outcome measures are varied and complex, there is no agreed taxonomy [9]. Hence, rehabilitation research will often use several measures. Secondly, this research will involve a multidisciplinary team. Finally, samples sizes are often small [10] since the range of disabilities is very extensive and diversity of conditions is high. Thus, rehabilitation research is often highly individualized to a small homogeneous group of people.

### Evidence in process evaluation research

The aim of a process evaluation is to understand the underpinning mechanisms that explain why an intervention works (or fails) [11, 12]. They are focussed on understanding how the characteristics of intervention components impact on its delivery and implementation to a set standard (MRC). Although in recent years process evaluations are becoming a common part of trial research proposals with an increased use in theories and frameworks driving and informing them (e.g. realist evaluation, normalization process theory), there is to date, limited guidance to help researchers design process evaluations [13, 14]. This is particularly true in the field of rehabilitation research. As a result, carrying out a process evaluation alongside a complex rehabilitation research trial can be seen as a daunting task, leading some researchers to discard the idea of embarking on one or organising them unsystematically [14].

To date, only one piece of guidance has been published about undertaking process evaluations, which was published whilst this research was underway [15]. The Medical Research Council (MRC) guidance aims at providing guidance about how to carry out process evaluations of public health interventions, and is considered by its authors as relevant to evaluating complex interventions. The guidance summarises why there is a need for process evaluations alongside current health research, and it then proposes a framework, which is highly informed by the MRC guidance on complex interventions [3]. It discusses process evaluation theory and then presents a practical section on how to carry out a process evaluation. The guidance covers issues of implementation, mechanisms of impact and influences and role of context. It also incorporates how the function and focus of a process evaluation will vary according to the stage at which is conducted and the particular type of complex intervention [16]. Each process evaluation will be different, but, the MRC guidance was created in order to facilitate its planning and conducting [13, 16]. According to several authors [17, 18] the tailoring of guidelines to particular contexts is of vital importance and can strongly influence their uptake by the end user. Rehabilitation research, as previously discussed, presents a particular set of challenges. Current guidelines such as the MRC guidance although relevant to complex interventions often do not address these challenges and therefore might present a number of limitations when applied to this context. This paper reports on the development of consensus guidelines that build on current ones and aim to solve their limitations tailoring their content to the individual challenges that define complex rehabilitation intervention research and its process evaluation.

## Methods

### Formal consensus – Study design

A formal consensus development process was undertaken by the researcher based on a modified nominal group technique (NGT) and informed by previous work carried out by Rycroft-Malone [19]. A formal consensus process was chosen over an informal one since it has been argued that guidelines produced as a result of informal consensus often formulate recommendations without drawing from research evidence [20]. Also, an informal process often follows unsystematic criteria and therefore resulting guidelines are not robust and can be highly subjective [19].

NGT is an interdisciplinary collaborative approach and this can work at enhancing the credibility of a guideline produced using this method. In other words, when end users of a guideline (in this case rehabilitation researchers) have been involved in its creation, this can have a positive influence on the future uptake of the guideline [19, 21, 22]. A number of strengths of this method have been identified. First, it allows for participants to discuss recommendations face to face, and, due to its highly structured nature, it can maximize the chances for all participants to contribute in an equal way [23]. Secondly, it is a technique that has been successfully used in the fields of health and rehabilitation research [24].

Participants were purposively sampled to reflect specialist knowledge and experience in rehabilitation research. Participants were asked to take part due to their status as ‘experts in rehabilitation and complex intervention research’. Invited participants worked in different universities in the United Kingdom and covered a range of demographic characteristics and career progressions. They qualified for selection based on their expertise on the matter under discussion [25] but also because they had the seniority in their field to implement the findings. The expert panel was expected to comprise 5–9 participants. Limited research in this area has shown that this range is appropriate, with less than 5 decreasing reliability and more than 9 causing coordination problems [26].

Ethical approval to carry out this work was obtained from the Coventry Research Ethics Committee (Reference: 09/H1210/88). Written and/or verbal informed consent to take part in this study was obtained from all participants.

#### Statements under consideration

The evidence available for these guidelines came from one source: the systematic review on the current state of process evaluation research in neurological rehabilitation research carried out by the main author [27]. This systematic review resulted in a number of provisional statements for carrying out process evaluations in neurological rehabilitation research. These statements were identified via the individual analysis and consequent overarching synthesis of two evidence streams: stream I, published process evaluations of neurological rehabilitation interventions and stream II, published guidelines and methodology on process evaluation. Stream I included 124 studies reporting on 106 interventions and stream II included 30 studies. The review concluded firstly, that there is a need for process evaluations to explore the role that intervention staff, their experience and set of skills play in the trial. Secondly, that it is vital for a process evaluation to address the nature and influence of context over time by monitoring staff’s learning effects and the possible impact on trial outcomes.

**Fig. 1:**
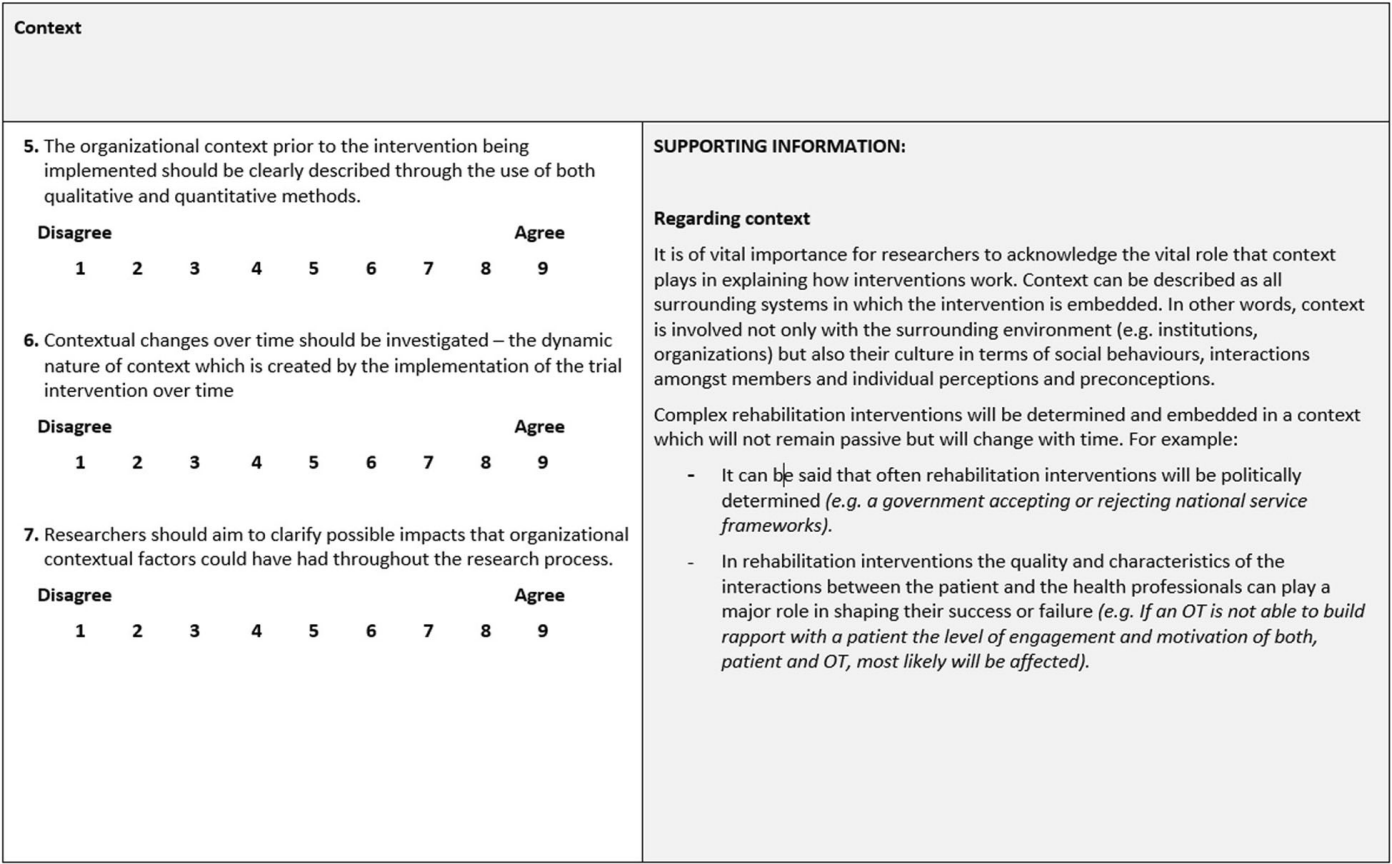
Example of interest area (context) including statements and supporting information

A total of 57 initial statements about process evaluation in rehabilitation research were identified. These 57 statements were grouped in 9 areas (Table 1) (for a complete list of statements please refer to Additional file 1). Each area was accompanied by a rationale providing a summary of the supporting information (Fig. 1 shows an example of one of these interest areas – context). The paperwork included explanations and supporting information for each of the areas under discussion.

**Table 1:** Number of statements per area of interest

Area of interest	*N* of statements
Complex interventions and theoretical approaches	4
Context	3
Recruitment	10
Description of intervention staff	4
Description of intervention	5
Preparing and assessing intervention staff	7
Delivery of the trial intervention	10
Understanding and interpreting process evaluation results	4
Methodology	10

#### Phase I - nominal group meeting

The nominal group meeting was organised following the standards reported by Rycroft-Malone [19]. In this meeting participants had the chance to discuss face to face, critique and rate each of the proposed statements (Additional file 1). Also, they could voice their opinions on the relevance of each of the suggested recommendations.

A suitable and convenient place for the meeting was chosen in order to increase the chances of participant’s availability. The lead author was the nominal group meeting facilitator. Prior to the meeting, all participants received via email a document including all statements to be discussed in the meeting (Additional file 1), and another document including a summary of the results from the systematic review [27]. Making this evidence available increased the chances of reducing bias as participants’ opinions are then influenced not only by their own personal experiences but also by the evidence provided [28].

Prior to the meeting, a participant information sheet was sent to all participants and written informed consent was obtained from all those attending the meeting. The complete meeting was audio recorded to assure that all information was captured. During the meeting, following a strict order, each of the 57 statements and supporting information were considered (Additional file 1). Firstly, participants were encouraged to discuss their opinions regarding the statement. Participants were then asked to privately rate the statement taking into account the research evidence, their expert opinion and the current state of rehabilitation research in this area of the UK. The participants were asked to rate the statement from 1 to 9 according to the following question: *How important is it for this statement to be included in the future guidelines?* This process was followed for the 57 statements allowing participants to take a break when necessary.

### Data analysis

Although there is no agreement on what is the best method to mathematically analyse this type of rating response [23, 28] the frequency of responses to each statement was calculated. For each statement, the median was calculated using SPSS for Windows. If the median score of the statement was 7–9 this meant that consensus had been reached and that the statement would be developed into the guidance recommendation. If the median was less than 2.99 then that would mean rejection of that statement. Finally, those statements with a median in the middle ground were retained for further discussion during telephone interviews and post nominal group meeting feedback (Phase II).

Data obtained from the audio-recording during Phase I was transcribed in full. In order to analyse this set of qualitative data a thematic analysis approach was taken following the method described by Braun and Clarke [29]. This method was chosen as it provides a rich and detailed account of the data whilst being flexible. First, the main author (PMA) re-read the transcription in order to gain familiarity with the data, which was then coded in order to capture conceptual meanings. Crosschecking by the co-authors was carried out with 10% of transcribed data to identify codes where there was lack of clarity. All codes were collated with their relevant data extracts. Themes were then identified as meaningful patterns across coded data.

#### Phase II - second round of feedback

Once results from Phase I were analysed a summary was emailed to all participants. This included a summary of main identified themes and a revised version of the proposed guidance recommendations according to the results from the nominal group meeting.

Phase II of the NGT involved telephone in-depth interviews will a set of expert participants in order to provide further feedback and critique the proposed revised version of the guidance recommendations. Verbal informed consent to take part in this study was obtained from all participants.

In line with ethical approvals, verbal informed consent was obtained from all participants and audio recorded at the start of each interview. Prior to the telephone interview, participants were asked to read the revised version of the guidelines. This allowed participants to see the spread of agreement and how their response related to the results from the group meeting. Certain items were selected for discussion with the focus primarily on statements where agreement had not been reached. These semi-structured telephone interviews focussed primarily on those statements that were the source of the most disagreement during Phase I. Participants were asked about both, the overall structure of the guideline and specific aspects such as the role of theory in informing process evaluations and issues around tailoring and context (for the interview schedule please refer to Additional file 2).

### Data analysis

All Phase II in depth interviews were transcribed in full; the same process as in Phase I was followed and thematic analysis was carried out following Braun and Clarke’s method [29]. Themes were identified and collated with those that emerged during Phase I. Finally, the main author, firstly independently and then, through discussion with the rest of the team members (co-authors), produced a final version of the guidelines which was in line with identified themes.

## Results

### Expert panel

The researcher contacted a total of 23 potential participants. 10 agreed to take part in this consensus work. Due to work commitments and difficulty timetabling mutually convenient dates, 5 out of the 10 participants attended the nominal group meeting (Phase I) and the remaining 5 participants took part in Phase II. Table 2 provides information regarding the professional characteristics of the participants and their involvement in the research process. 5 of the participants were professors in their field and therefore had high level of expertise. Two of the participants were working towards completing their PhD studies. Participants’ backgrounds were varied; one was a physiotherapist, three nurses, one an exercise physiologist, one a speech pathologist, one a psychologist and two were medical doctors.

**Table 2:** Professional characteristics and involvement of members of the consensus expert panel

Current research role	Background	Phase I	Phase II
Professor of Clinical Biostatistics	Biostatistics	√	
Doctoral Research Fellow	Speech pathology and therapy	√	
Professor of Stroke and Older People’s Care	Nursing	√	
Honorary Research Associate	Nursing	√	
Senior Research Fellow	Nursing	√	
Professor in Exercise Physiology	Exercise physiology		√
Reader in Psychology	Psychology		√
Clinical Senior Lecturer	Medical sciences		√
Professor of Stroke Medicine	Medical sciences		√
Research Officer	Physiotherapy		√

**Fig. 2:**
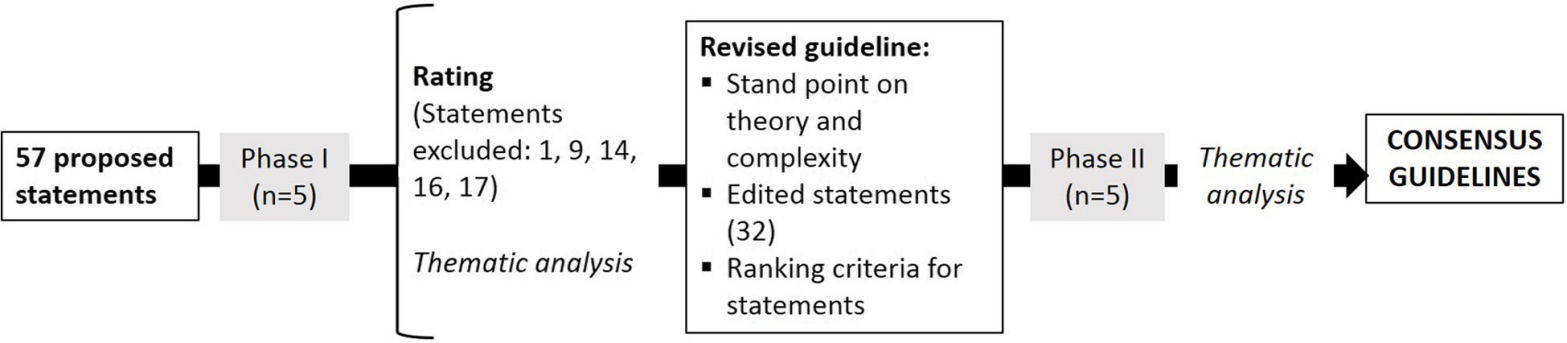
Formal consensus process

The results of the ratings were calculated for each of the statements. The median value for the statement together with the highest score and lowest score were calculated. 5 statements (n.1, n.9, n.14, n.16 and n.17) were excluded since consensus was not reached. The remaining 53 statements met the criteria to be included in the guidelines; however, participants expressed these needed further editing, clarifying and grouping in order to reduce the number of recommendations. As a result of the formal consensus process (Fig. 2), the initial 57 proposed statements were edited in order to incorporate comments and feedback from participants. These edits included changes in the use of terminology and in the order and grouping of the statements as well as general corrections to increase the clarity of the language. In addition, this revised version included an introduction section stating the underlying *standpoint* of the researchers regarding the nature of complexity.

Four themes were identified during the formal consensus process as having a significant influence on participants’ ways of thinking at the time of discussing statements and the need for them to be included in the proposed guidelines (Table 3). The data gathered during both phases was key in order to understand what the rehabilitation research community think about process evaluations. Participants openly discussed issues around the practicalities and the challenges of process evaluation research. This consensus work became a platform for researchers to voice their understanding about what is and what should be the aim of a process evaluation. Table 3 provides a summary of themes that were identified during the formal consensus process. These themes describe a number of issues in regards to the guidance and its potential use for rehabilitation researchers which participants suggested needed addressing.

**Table 3:** Identified themes across Phase I and II

Theme	Description
The practicalities of doing research – being realistic about what ‘can be done’	All participants agreed that there is a degree of compromise which impacts on what can realistically be achieved at the time of evaluating processes. Participants expressed their desire to not only rate recommendations in terms of the need for them to be included in the guidelines, but also to rank these statements in terms of their relative importance.
*Stand point* – role of theory, concepts and roles	Participants expressed how it is important for any guidelines to include an explanation of the assumptions that underpin it. The participants’ epistemological and ontological stance highly influenced their views regarding proposed recommendations and their understanding of the guidelines’ content. Likewise, participants expressed different views in regards of the role that theory plays at the time of designing and carrying out a process evaluation. Participants considered that for guidelines to work, they need to clearly explain their underlying assumptions. In this way, the rehabilitation researcher can make an informed decision at the time of following the proposed guidelines.
Investigating *tailoring* and ‘making connections’	Participants identified the need for a process evaluation to investigate the level of tailoring and its impact on outcomes. They discussed in depth the challenges in assessing the degree of tailoring taking place at the time of trialling a rehabilitation intervention. Participants widely agreed on the fact that in the everyday running of a trial it was unrealistic to assume complete consistency in the way professionals deliver proposed rehabilitation interventions.
Who is the end user?	Participants unanimously agreed on the fact that all process evaluations should have clear aims and objectives and that these would differ according to the type of trial under evaluation and the timing of the evaluation. The proposed guidelines need to state who the end users are; rehabilitation researchers will then be responsible for tailoring its recommendations to best fit their evaluation aim. Participants agreed that the process evaluation guidelines would need to be tailored, not only to a particular process evaluation, but also to end users’ needs.

#### The consensus guidelines

The guidelines for carrying out process evaluations within complex interventions rehabilitation research were produced (Table 4) from findings from Phase I and Phase II. The proposed guidelines include a number of clarifying points in regards to: firstly, who are the guidelines’ target audience and how they should be used and adapted by rehabilitation researchers according to the design, type and the timing of the trial under evaluation. Secondly, a brief explanation clarifying the underlying assumptions underpinning the consensus guidelines and linked recommendations. Finally, seven sections in which the recommendations are grouped. These sections represent different aspects or stages, which the rehabilitation researcher will face throughout the evaluation process. The following describes the domains including an illustrative example for each.

- *Theoretical work*: addressed issues in relation to the theoretical underpinnings of the trialled intervention. Researchers are guided to review the theoretical underpinnings not only of the rehabilitation intervention but also the implementation approach. For example, Byng et al. [30] carried out the process evaluation of an intervention to improve primary healthcare for patients with long-term mental illness following a realist evaluation approach. They reported that through realist evaluation the team was able to identify the interactions taking place, not only between intervention components, but also with the embedded external context.
- *Design and methods*: this describes a number of steps aimed at treating a process evaluation as a piece of research in its own right. Researchers are advised to provide a clear definition of chosen process evaluation terminology, define clear aims and objectives and provide a detail description of selected data collection methods and timings. Finally, the guidelines recommend researchers addressing the interactions between process and outcome measures. For example, a number of protocols for process evaluations have been published alongside the main trial’s protocol [31, 32].
- *Context:* this section addresses the importance of understanding and accounting for contextual factors, their role and their potential impact on process and outcomes over time For example, the process evaluation of a randomized controlled trial (RCT) looking at the benefits of a programme for caregivers of inpatients after stroke (TRACS study) [33]. This evaluation investigated the impact that contextual factors had during the process of embedding the intervention into the routine practice of a stroke unit. The researchers explored in detail contextual factors such as organisational history and policies, team relationships, responsibility sharing and staff engagement.
- *Recruitment and retention*. The process evaluation should review the outcome evaluation’s recruitment and retention procedures in order to identify potential barriers and facilitators. It should also clearly describe the strategies and criteria informing the recruitment of participants into the process evaluation. Scianni et al. [34] reviewed in detail their recruitment procedures and identified transport to and from the health setting as the main barrier to participation in a trial investigating the impact of gait training for stroke survivors.
- *Intervention staff*. This section firstly addresses the need to investigate the characteristics of staff in charge of delivering the intervention and identify how these can potentially have an effect on intervention implementation and impact. Secondly, it recommends the process evaluation to review the training provided to intervention staff in order to identify possible impact on outcomes. For example, Chung [35], in his study assessing the impact of a reminiscence programme for older adults with dementia provided a detailed description of the training component and expected learning outcomes. Intervention staff’s knowledge on delivering the programme was assessed using quizzes and questionnaires.
- *Delivery of the intervention*. The guidelines recommend that process evaluation researchers should focus on tailoring and investigate the strategies in place in order to guide it and measure it. In addition, researchers should investigate barriers and enablers to implementation by reviewing strategies in place to improve or support the fidelity of the rehabilitation intervention. The process evaluation should review strategies in place to measure ‘dose delivered’ and ‘dose received’. Finally, participant’s experiences and acceptability of the intervention should be investigated. To date, it is rare for research studies to provide intervention providers with clear guidance on how to assess which is the ‘right amount’ of tailoring [27]. However, studies such as Mayo et al. [36] set an example by investigating how an exercise programme post-stroke was tailored to patients needs whilst keeping to the protocol guidelines.
- *Results*. This section addresses the need to describe in detail the synthesis of process and outcome evaluation results. This synthesis should be informed by the theoretical underpinnings behind both, the outcome evaluation and its implementation. For example, in their study looking at a rehabilitation intervention for adults with brain injury, Letts and Dunal [37] developed a logic model through consensus work, which integrated information on process and outcomes.

**Table 4:** Guidelines for carrying out process evaluations within complex rehabilitation interventions research

Section	No	Recommendation
Theoretical work		
	1.1	Review and state the theoretical underpinnings of the rehabilitation intervention under investigation
	1.2	Review and state the theoretical underpinnings of the implementation approach of the rehabilitation intervention under investigation
	1.3	Describe in depth the structure of the rehabilitation intervention in terms of its components and their potential interactions
Design and Methods		
	2.1	Provide a clear definition of chosen terminology (e.g. adherence, fidelity, integrity etc.)
	2.2	Have a defined scope and clear aims and objectives - a process evaluation protocol should be produced
	2.3	Clearly describe and justify the use of a set of measures and evaluation criteria for the process evaluation
	2.4	Provide a detail description and justification of selected process evaluation data collection methods
	2.5	Clearly explain and justify chosen timings for process evaluation data collection
	2.6	Collect relevant/appropriate data from both intervention and control sites
	2.7	Use a variety of methods and strategies to gather data, including both qualitative and quantitative approaches
	2.8	Should aim at publishing its results alongside outcome evaluation results (in order to reduce the chance of biases)
	2.9	Address the interactions between process and outcome evaluations *(*e.g. *researchers should decide if they take the risk of threatening the outcome evaluation* via *evaluating processes or if they accept that there will be tailoring which can be guided through the process evaluation)*
Context		
	3.1	Clearly describe and investigate contextual factors and their potential impact on the process and outcome evaluation. *The role of context in shaping both implementation (*e.g. *how it’s done) and impact (whether it works) should be clearly investigated*
	3.2	Account for the dynamic nature of context - investigate contextual changes and their potential impact on the process and outcome evaluation over time
Recruitment and Retention		
	4.1	Review the outcome evaluation’s recruitment procedures in order to identify potential recruitment barriers and facilitators
	4.2	Review the strategies that the outcome evaluation has in place to maximize participant retention levels
	4.3	Clearly describe the strategies and criteria informing the recruitment of participants into the process evaluation
	4.4	Investigate the barriers and facilitators to the recruitment of participants into the process evaluation
Intervention staff		
	5.1	Review the characteristics of the outcome evaluation intervention staff (e.g. level of skill, experience, number, demographics, motivations and perceptions regarding the outcome evaluation) and identify those potentially impacting on intervention delivery and impact
	5.2	Review the training provided to intervention staff in order to identify possible impacts on outcomes. Explore issues such as: *does the training define a performance criteria and set of goals to achieve? Is skill acquisition/competence of intervention staff assessed post training? Does the training include systems in place in order to maintain and support staff’s skills over time?*
	5.3	Review the outcome evaluation’s strategies in place to assess competence of intervention staff over time in order to identify possible learning curve effects
Delivery of the intervention		
	6.1	Investigate any strategies in place in order to guide, inform and measure the tailoring of the outcome evaluation intervention
	6.2	Review and assess the quality of any implementation strategies to improve/support the fidelity of the proposed intervention.
	6.3	Investigate, in detail, barriers and enablers to the implementation and delivery of the intervention and evidence surrounding the chances of implementation failure
	6.4	Review the strategies in place in order to measure the ‘dose delivered’
	6.5	Review the strategies in place in order to measure the ‘dose received’
	6.6	Investigate in detail participants’ experiences and acceptability of the intervention
Results		
	7.1	Describe in detail the synthesis of process evaluation and outcome evaluation results
	7.2	The theoretical underpinnings behind both, the outcome evaluation intervention and its implementation should inform the explanations and the synthesis of process and outcome evaluation results

It is strongly recommended to consider these guidelines alongside recommendations on reporting outcome evaluations (e.g. CONSORT statement)

These guidelines are of use to researchers carrying out research on complex rehabilitation interventions and the recommendations will need to be considered and adapted accordingly depending on the research stage/phase or type of study (e.g. feasibility trial, main trial, etc.)

These guideline recommendations build on the following assumptions about the nature of complexity in complex intervention rehabilitation research:

- Complex rehabilitation interventions are those made up of a number of components, which interact with each other, and with patient and other factors to bring about changes in patient outcomes

- The impact of complex interventions is greater than the sum of the effects of their component parts, and is a product of both the changes embedded in both the intervention hypotheses and the implementation approaches used. In other words, and in order to provide explanations of how a complex intervention works, for who and under what circumstances, this guideline considers that outcome evaluation and process evaluation are inextricably linked

## Discussion

This paper presents a set of consensus guidelines for carrying out process evaluations within complex rehabilitation research. These guidelines allow sufficient flexibility in order to be adapted accordingly depending on the research design and study type and they work on the assumption that complex rehabilitation interventions are those made up of a number of components, which interact with each other to bring about changes in outcomes. Furthermore, these guidelines consider that the impact of the complex intervention is greater than the sum of the effects of their component parts and is a product of not only the changes embedded in the intervention hypothesis but also the implementation approaches informing it [38]. The aim of these guidelines is to update and contribute to the published evidence by extending its coverage to rehabilitation research, its processes and theoretical underpinnings. These guidelines provide a new lens for rehabilitation researchers attempting to carry out a process evaluation and they build on published work such as the UK MRC guidance [15] in an attempt to address the difficulties and challenges faced, in particular, by those researchers dealing with complex rehabilitation interventions. For example, one of these challenges is in regards to participant recruitment into rehabilitation trials which often follows a criteria that is therapeutically based and therefore more complex, instead of based on a screening tool [39]. The proposed guidelines acknowledge this and propose a number of recommendations that guarantee the close exploration of the trial’s recruitment procedures in order to identify potential barriers and facilitators and their impact on outcomes. Furthermore, these guidelines recommend in depth review of the strategies implemented during the outcome evaluation in order to maximise participant retention (e.g. transportation to and from research base). A further challenge faced by rehabilitation researchers planning an RCT is making sure that treatment differentiation is kept throughout the study. This can be extremely hard considering the role that tailoring often plays throughout the delivery of the trialled intervention. The proposed guidelines address this challenge by advising on the need to firstly, investigate strategies to guide, inform and measure the tailoring, and secondly, assess the quality of any implementation strategy aimed at improving or supporting the fidelity of the rehabilitation intervention. Finally, these guidelines understand the further challenges that rehabilitation trials face in terms of recruiting intervention staff. The skills, previous experience and knowledge of those administering the intervention can influence intervention impacts [7]. This issue is particularly addressed in these guidelines with a number of recommendations focussing on what the process evaluation should investigate in regards to intervention staff characteristics, training provided and possible impact on outcomes.

In these guidelines, outcome evaluation and process evaluation are considered to be inextricably linked. With this in mind, these guidelines work towards a consensus in regards to how rehabilitation researchers should go about carrying out process evaluations and how this evaluation should be linked into the proposed trials. Additionally, these guidelines are innovative, in addressing the importance of learning effects and contextual changes with time, when evaluating the processes that take place as part of a research trial. Finally, the guidelines here presented stress the vital importance of describing in detail the components of the rehabilitation interventions and their interactions. This demand is in line with the requirements of other highly accepted published tools such as The Consolidated Standards for Reporting Trials (CONSORT) 2010 statement [40] or the more recent Template for the Intervention Description and Replication (TIDieR) checklist and guide [41].

As the data here presented shows, researchers are aware of how their decisions in terms of process evaluation will be closely influenced by the type and stage of the study. As put by Moore et al. [16], “*the focus of process evaluation will vary according to the stage at which it is conducted”* (p.2). Thus, in line with what other authors [13, 42] have stated, the guidelines here presented will need to be tailored to rehabilitation researchers’ particular needs, since there is no single way to carry out a process evaluation. Issues around the design, the phase, the timing of the study or a number of contextual factors will play a major role at the time of designing and carrying out a process evaluation. Furthermore, as expressed by Moore et al. [16], even when the feasibility trial has been under a process evaluation, there will still be the need to carry out another one, alongside the full trial, because it is likely that the intervention, and this is particularly true for rehabilitation interventions, will face new problems and new challenges will emerge when implementing at a larger scale. Finally, the guidelines here presented incorporate the idea that changes in contextual factors, responsible for triggering intervention mechanisms [43], are likely to take place throughout the research period and will therefore need to be addressed by the process evaluation.

One of the challenges faced by rehabilitation researchers planning an RCT is making sure that treatment differentiation is kept throughout the study. In addition to this, several authors [44, 45] have identified addressing ‘the science of client centred replication’ as a major challenge for today’s health care research. Thus, it is of vital importance to address the issue of tailoring of the intervention if the researcher aims to investigate its fidelity in depth [46, 47]. The proposed guidelines address this need by advising on the need to firstly, investigate strategies to guide, inform and measure the tailoring, and secondly, assess the quality of any implementation strategy aimed at improving or supporting the fidelity of the rehabilitation intervention. In this way, and in answer to a need that has been previously identified by several authors [13, 14], these guidelines allow for sufficient flexibility and room for manoeuvre in order to be tailored to the type of intervention and the type of study design, whilst facilitating standardisation of research practice. Furthermore, these guidelines are in tune with the challenges that rehabilitation trials face in terms of recruiting intervention staff. The skills, previous experience and knowledge of those administering the intervention can influence intervention impacts [7]. This is particularly addressed in these guidelines with a number of recommendations focussing on what the process evaluation should investigate in regards to intervention staff characteristics, training provided and possible impact on outcomes.

The data here presented show, and as it has been discussed in the literature [16], that there are arguments for both the separation and the integration of process evaluation and outcome evaluation teams. These guidelines assume some integration between outcome and process evaluation. The guidelines we here propose consider that data on implementation should be integrated into the analysis of outcomes and that emerging process issues identified in the process evaluation should be integrated into trial data design and collection. Also, the authors understand that by considering outcome and process evaluation to be inextricably linked, the rehabilitation researcher might avoid duplication of efforts and reduce the burden on participants at data collection stages. As raised by O’Cathain et al. [48], effective integration and addressing the links between process and outcome evaluations will take place only when members of both teams value each other’s contribution and when the principal investigator understands and agrees with the value of integration. Closely linked to this, authors such as Audrey et al. [49] have identified that one of the main challenges of implementing process evaluation within clinical trials is the overlapping roles within the team and distinguishing between the intervention and its evaluation. The proposed consensus guidelines support the need for close integration of process and outcome evaluations.

The modified consensus NGT method [19], used in the creation of this guideline, proved to be straightforward. The nominal group meeting was demanding upon participants because there were a large number of recommendations to discuss. Also, it was hard for the researcher to judge how successfully ‘group dynamics’ were controlled and how much the personality and compliance of the participants impacted on the cooperation of the panel of experts. However, there are a number of additional strengths in this piece of work. This consensus work provided an opportunity for the researcher to be involved in collaborative working amongst a number of rehabilitation researchers from a number of different disciplines. Finally, as Rycroft-Malone [19] points out, the use of a collaborative approach, by listening to experts in the field, could have a positive impact on the ultimate uptake of the guideline as it is seen as being more credible.

### Limitations

The number of participants who took part in both phases of the consensus work was lower than originally anticipated. However, all participants were highly experienced in carrying out rehabilitation research and were all academics. The statements under consideration during the consensus process were drawn from a systematic review that focussed on neurological rehabilitation and a small number of experts in the panel had a neurological research background as well. This neurological focus could have influenced the outcome of this consensus work. Finally, all expert participants were based in the UK and are likely to be more familiar with the challenges and nuances of the British healthcare research context. The authors understand firstly, that further work will be required to test the usefulness and applicability of the proposed guidelines to the work that rehabilitation researchers are currently undertaking not only in the UK but internationally. Secondly, that it is likely that these guidelines will be read and used by those researchers who share its underpinning assumptions in regards to the nature of complex interventions.

## Conclusions

This paper has outlined the process of the development of new consensus guidelines for designing and carrying out process evaluations of rehabilitation intervention trials. The aim of these guidelines is to update and contribute to the published evidence by tailoring its coverage to the particular challenges that define rehabilitation research, its processes and theoretical underpinnings. The results here presented break new ground in terms of concepts and theory and work towards a consensus in regards to how rehabilitation researchers should go about carrying out process evaluations and how this evaluation should be linked into the proposed trials. Although these guidelines are written from the perspective of researchers with experience of carrying out trials of complex rehabilitation interventions, it is also relevant and useful to stakeholders from other research domains such as funding agencies, when making decisions regarding allocation of funding.

## Additional files

Additional file 1: Statements for consensus group. (PDF 358 kb)

Additional file 2: Interview schedule. (PDF 459 kb)

## Abbreviations

MRC: Medical research council
NGT: Nominal group technique
RCT: Randomized controlled trial
