# Supplementary material for: The generation of consensus guidelines for carrying out process evaluations in rehabilitation research
Source: BMC Med Res Methodol. 2018 Dec 29;18:180. doi: 10.1186/s12874-018-0647-y (PMC6311071; doi:10.1186/s12874-018-0647-y)
Supplement: Supplementary file 1 — Statements for consensus group. (PDF 358 kb) [file 12874_2018_647_MOESM1_ESM.pdf]

Additional file 1_Masterson-Algar et al. 2018

Complex interventions and theoretical approaches

1. There should be a clear description of the theoretical base behind the structure and delivery of the neurological rehabilitation intervention (e.g. a way to do this could be by process evaluations testing the validity and usefulness of proposed theoretical rehabilitation frameworks)

Disagree								Agree
**1**	**2**	**3**	**4**	**5**	**6**	**7**	**8**	**9**

2. The structure of the neurological rehabilitation intervention should be clearly described in terms of its components

Disagree								Agree
**1**	**2**	**3**	**4**	**5**	**6**	**7**	**8**	**9**

3. Process evaluations should draw on methodological guidance

Disagree								Agree
**1**	**2**	**3**	**4**	**5**	**6**	**7**	**8**	**9**

SUPPORTING INFORMATION:

Rehabilitation interventions are often complex:

Complex interventions are defined as those made up of a number of components or active ingredients that interact with each other and with outside factors to bring about changes to outcomes. It is important to be clear regarding what is 'complicated' and what is 'complex': complicated problems are formed of a number of parts that can be solved and their functioning can be predicted by using identified formulae and instructions; complex problems however rarely benefit from these tools, since they are uncertain. Complex problems are solved allowing time for learning about each component and for making sense of events taking place. Thus, the evaluation of complex interventions represents a great challenge since their path to success is variable and cannot be accurately predicted. Crucially, the difficulty in defining in detail rehabilitation treatments in terms of what are their 'active ingredients' and what is their impact is very challenging. Most of rehabilitation interventions will have several active ingredients.

Complex rehabilitation interventions can often be:

- Offered multiple times to multiple participants that can belong to a number of different groups.
- Complex behavioural treatments to the contrary of passive or surgical treatments.
- Delivered in a personal way where interactions therapist/patient play a significant role.
- Tailored to patient's needs at the time of defining goals or treatment plans.
- Designed in a number of sessions to allow time for individuals to learn and comprehend its content.
- Delivered in different locations and sites which can change.
- Delivered to individuals, families, combinations, etc.
- Delivered to individuals who are not 'passive recipients' of the intervention, individuals who will perceive and take on board the intervention in their own unique manner (for

**4.** There should be a clear explanation of how the methodological guidance is applied to the process evaluation (e.g. if a guidance is chosen it is necessary to clearly explain how was the guidance followed and how did the process evaluation remain in line with the guidance's proposed frameworks/steps)<br><br>**Disagree** **Agree**<br><br>**1 2 3 4 5 6 7 8 9**	example individuals will decide how intensively they want to get involved in the intervention)<br>- Furthermore rehabilitation research is often **context specific** and defined as the interaction between the individual and the environment. In other words, identifying contextual processes (physical, psychological, social, etc.) and acknowledging that researchers bring their values into situations is of great importance when thinking about the science of rehabilitation. Therefore, researchers working in this field need to design strategies and ways to explore and measure context.<br><br>It is feasible to describe an intervention in terms of its 'active ingredients'. However, throughout the research process the intervention should be seen *as a whole which is greater than the sum of its parts.* Reducing the complex intervention to a number of components and understanding how these work individually might make the intervention loose its essence. Understanding how parts of the intervention work should always be considered in close relation to how the intervention works as a whole.<br><br>**Theories and rehabilitation**<br><br>Rehabilitation professionals share assumption regarding firstly, the nature of their work: they need to be apolitical, relevant and useful. Secondly, the nature of their goals: to increase function, independence and quality of life and finally, the nature of the relationship with the client, which has to be holistic and client-centred. The problem is that these theoretical assumptions so far lack in evidence base support. Many areas of rehabilitation are underdeveloped from a theoretical perspective and energy should be invested, as it is spend in empirical research, in developing well-articulated theories and consequent theoretical models. The theory behind the structure and delivery of a proposed rehabilitation intervention will need to reflect its complexity and address it.<br><br>What is the 'theory of change' behind the proposed rehabilitation intervention? How many theories are needed to guide rehabilitation research, or should there be an overarching one? Many theories appear relevant to rehabilitation, for example learning theories, theories of goal setting, theories related to self-management and also theories looking at changes at the person-environment interface such as theories of diffusion of innovation.

**Context**	
**5.** The organizational context prior to the intervention being implemented should be clearly described through the use of both qualitative and quantitative methods.<br>**Disagree** **Agree**<br>**1 2 3 4 5 6 7 8 9**<br><br>**6.** Contextual changes over time should be investigated – the dynamic nature of context which is created by the implementation of the trial intervention over time<br>**Disagree** **Agree**<br>**1 2 3 4 5 6 7 8 9**<br><br>**7.** Researchers should aim to clarify possible impacts that organizational contextual factors could have had throughout the research process.<br>**Disagree** **Agree**<br>**1 2 3 4 5 6 7 8 9**	**SUPPORTING INFORMATION:**<br>**Regarding context**<br>It is of vital importance for researchers to acknowledge the vital role that context plays in explaining how interventions work. Context can be described as all surrounding systems in which the intervention is embedded. In other words, context is involved not only with the surrounding environment (e.g. institutions, organizations) but also their culture in terms of social behaviours, interactions amongst members and individual perceptions and preconceptions.<br>Complex rehabilitation interventions will be determined and embedded in a context which will not remain passive but will change with time. For example:<br>- It can be said that often rehabilitation interventions will be politically determined (e.g. a government accepting or rejecting national service frameworks).<br>- In rehabilitation interventions the quality and characteristics of the interactions between the patient and the health professionals can play a major role in shaping their success or failure (e.g. If an OT is not able to build rapport with a patient the level of engagement and motivation of both, patient and OT, most likely will be affected).

Recruitment

**8.** Process evaluations of neurological rehabilitation research studies should clearly describe the trial's recruitment procedures.<br><br>**Disagree** **Agree**<br>**1 2 3 4 5 6 7 8 9**<br><br>**9.** Reasoning behind participants being recruited for the trial should be provided (e.g. excluding patients with cognitive impairment is often the case. The rationale behind this decision should be clearly explained considering the considerable prevalence of stroke survivors having a cognitive impairment)<br><br>**Disagree** **Agree**<br>**1 2 3 4 5 6 7 8 9**<br><br>**10.** Barriers and facilitators to recruitment for the trial should be clearly investigated.<br><br>**Disagree** **Agree**<br>**1 2 3 4 5 6 7 8 9**	**SUPPORTING INFORMATION:**<br><br>**Recruitment in rehabilitation research** can present a number of difficulties and challenges, both at an individual and at an organizational level.<br><br>For example:<br><br>- At present rehabilitation practice lacks of a nationally standardized and accepted set of outcome measures, therefore researchers often have to use and individual and trial/specific screening tools in order to identify and assess the suitability of the participants. Having a recruitment criteria that is therapeutically based is a more complicated procedure and therefore is more expensive and time consuming that the recruitment process in medical trials who often utilise a simple chart review.<br>- Rehabilitation researchers often have to give special attention to retention due to the nature of the patients, for example, their recruitment budget will often need to include cost of participants transportation to and from the research base or 'reminding methods' such as postcards or phone calls.<br>- It is often hard to reach patients who are not registered as being part of rehabilitation services. The recruiting effort will be considerable and often needs to use other alternative sources and venues which can be time consuming and costly.<br>- Recruiting effort will need to account for the characteristics of this group of service user who will often have mobility and/or cognitive difficulties which might have led to limited social involvement and very little time spent out of his/her home.

**11.** Strategies to recruit participants to the process evaluation should be clearly described.<br><br>**Disagree** **Agree**<br>**1 2 3 4 5 6 7 8 9**<br><br>**12.** Criteria for selecting participants for the process evaluation should be clearly identified.<br><br>**Disagree** **Agree**<br>**1 2 3 4 5 6 7 8 9**<br><br>**13.** Barriers and facilitators to recruitment of participants into the process evaluation should be investigated.<br><br>**Disagree** **Agree**<br>**1 2 3 4 5 6 7 8 9**<br><br>**14.** Process evaluations should investigate measures in place to attract participants and encourage them to remain involved in the trial.<br><br>**Disagree** **Agree**<br>**1 2 3 4 5 6 7 8 9**	

15. The involvement of participants recruited for the process evaluation should be monitored.

Disagree **Agree**

1 2 3 4 5 6 7 8 9

16. Process evaluations of clustered trials should clearly describe the site recruitment procedure in place (e.g. minimum quality standards, funding, incentives).

Disagree **Agree**

1 2 3 4 5 6 7 8 9

17. How withdrawal from sites was carried out should be clearly explained.

Disagree **Agree**

1 2 3 4 5 6 7 8 9

**Description of intervention staff**	
**18.** A detail description of who (and how many) delivered the neurological rehabilitation intervention should be given.<br>**Disagree** **Agree**<br>**1 2 3 4 5 6 7 8 9**<br>**19.** Intervention staff previous relevant experience and skills should be recorded.<br>**Disagree** **Agree**<br>**1 2 3 4 5 6 7 8 9**<br>**20.** Motives for the participation of intervention staff in the study should be explored.<br>**Disagree** **Agree**<br>**1 2 3 4 5 6 7 8 9**<br>**21.** Intervention staff perceptions regarding the research study and possible impacts of the intervention should be investigated.<br>**Disagree** **Agree**<br>**1 2 3 4 5 6 7 8 9**	**SUPPORTING INFORMATION:**<br>Neurological rehabilitation interventions often require a level of skill and understanding of different techniques and methods. They often involve treating complex patients with complex needs. Thus, it is vital to have a good understanding of the characteristics of all staff responsible for delivering the neurological rehabilitation intervention. Staff's previous experience and level of skill will potentially have an impact on the way the intervention is being delivered and also on the way this intervention will bring about changes to outcomes.

Description of intervention

22. The study intervention should be detailed in a protocol/manual.

Disagree								Agree
**1**	**2**	**3**	**4**	**5**	**6**	**7**	**8**	**9**

23. All structures and processes involved in the intervention should be fully described.

Disagree								Agree
**1**	**2**	**3**	**4**	**5**	**6**	**7**	**8**	**9**

24. The protocol should state how much tailoring and flexibility of the intervention is allowed.

Disagree								Agree
**1**	**2**	**3**	**4**	**5**	**6**	**7**	**8**	**9**

25. A guide for tailoring should be provided to all professionals implementing the intervention.

Disagree								Agree
**1**	**2**	**3**	**4**	**5**	**6**	**7**	**8**	**9**

26. The degree of tailoring should be investigated within the evaluation.

Disagree								Agree
**1**	**2**	**3**	**4**	**5**	**6**	**7**	**8**	**9**

SUPPORTING INFORMATION:

Tailoring rehabilitation interventions

The **'science of replication'** in rehabilitation research requires further development. There is more to delivering a rehabilitation intervention than just measuring how many elements were delivered. Rehabilitation research should avoid a 'cookbook' approach if it intends to understand the vital role played by contextual factors. As a result there is an increased awareness of the need to tailor rehabilitation interventions to patients' needs and cultural background in order to increase their potential to be effective. To be able to replicate a rehabilitation intervention across different settings it will be necessary to adapt it (tailor it) to some extent and this is likely to create tension between the need to tailor and the need to maximise treatment integrity.

Tailoring should not mean intervention staff 'improvising as they go along', it should mean that what is standardized will be contrasted and clearly defined and monitored against what is customized. As a result, the assessment of how the rehabilitation intervention was administered according to the plan will have to be standardized and tailored to the actual level of standardization and tailoring of the trialled intervention. Succeeding at this can be extremely challenging for rehabilitation researchers. A first vital step could involve identifying and recording the delivery of unplanned components (for example using specific recording sheets). This information can help for example, to identify which aspects need to be included in the re-training of intervention staff on the requirements to follow the protocol. It can also help identify aspects of the intervention which need modifying.

Preparing and assessing intervention staff

27. The training provided to intervention staff involved in the research should be clearly described (e.g. details on when and where will the training take place, who needs to attend, who will deliver it, etc.)

Disagree **Agree**

1 2 3 4 5 6 7 8 9

28. Training provided should have a defined set of goals to achieve.

Disagree **Agree**

1 2 3 4 5 6 7 8 9

29. There should be well-defined performance criteria associated with the intervention.

Disagree **Agree**

1 2 3 4 5 6 7 8 9

30. Skill acquisition/competence of intervention staff should be measured post training as the basis for participating in the study.

Disagree **Agree**

SUPPORTING INFORMATION:

Training staff to provide a rehabilitation intervention

It is widely accepted that training the staff responsible for the implementation of the trialled rehabilitation intervention is beneficial:

- Through training and supervision you can refine the work of the providers who in most cases will already have experience in this trialled intervention.
- The training can help teach the provider to not use their usual approaches if they are not part of the intervention - staff should familiarize themselves with the trial's manual/protocol during the training.
- Training provides a chance to discuss the philosophy underlying the intervention.
- Training will give a chance to intervention providers to practice the necessary skill set.

In rehabilitation trials it should be feasible to assess professionals' skills prior to the start of the trial. However, training staff involved in rehabilitation trials is often ongoing in order to assure that skills are maintained over time. In such cases an initial skill assessment could not be used as a basis for participation but regular/periodical assessments could be the solution.

**1 2 3 4 5 6 7 8 9**<br>**31.** Competence of intervention staff should be monitored over time in order to identify learning curve effects.<br>**Disagree** **Agree**<br>**1 2 3 4 5 6 7 8 9**<br>**32.** Methods should be in place in order to maintain skills over time (e.g. re-training, supervision, peer support, online notice boards, etc.)<br>**Disagree** **Agree**<br>**1 2 3 4 5 6 7 8 9**<br>**33.** Any additional implementation strategies to improve/support the fidelity of the intervention should be evaluated (e.g. performance evaluation).<br>**Disagree** **Agree**<br>**1 2 3 4 5 6 7 8 9**	Staff involved in delivering a rehabilitation intervention will learn overtime and they will become more familiarized with the techniques, patient characteristics, organizational contexts etc. Therefore, investigating staff learning curves throughout the trial and how these might explain trends in outcomes would be highly beneficial. It is equally important to have measures in place to assess how intervention staff have maintained skills over time.

<table>
<tr><td colspan="2">Delivery of the trial intervention</td></tr>
<tr><td>
34. Process evaluations should investigate barriers and enablers to the implementation of the intervention.

Disagree Agree
1 2 3 4 5 6 7 8 9

35. Process evaluations should clearly define quantitative indicators that reflect acceptable adherence to the intervention dosage across constituent components.

Disagree Agree
1 2 3 4 5 6 7 8 9

36. Process evaluations should clearly define what strategies were in place in order to measure ‘dose delivered’.

Disagree Agree
1 2 3 4 5 6 7 8 9

37. There should be well defined strategies in place to be able to measure ‘dose received’.

Disagree Agree
1 2 3 4 5 6 7 8 9
</td><td>
SUPPORTING INFORMATION:

Whilst for example in drug trials the delivery of the intervention is relatively simplistic in the case of rehabilitation interventions it often is not. For example, in the case of rehabilitation the accurate delivery of the intervention can be highly dependent on for example:

- The level of skill, previous experience and knowledge of the intervention staff - possible biases and previous experience can influence or clash with intervention
- Individual characteristics of patients beyond the intervention (e.g. depression, cognitive impairment, acceptance or attitude towards the intervention, personal factors, geographical factors, etc.). Heterogeneity of trial participants will be likely even after detailed screening according to inclusion and exclusion criteria.
- The difficulty with blinding, participants will know, in most cases, the intervention they are receiving.
- Difficulty with assessing participants understanding of the purpose of the intervention (for example when participants have some level of cognitive impairment which is often the case in rehabilitation research).
</td></tr>
</table>

**38.** Process evaluations should clearly define quantitative and qualitative indicators that reflect acceptable quality in the delivery of the study intervention.<br>**Disagree** **Agree**<br>**1 2 3 4 5 6 7 8 9**<br><br>**39.** Process evaluations should clearly explain the strategies in place in order to assess quality of intervention implementation.<br>**Disagree** **Agree**<br>**1 2 3 4 5 6 7 8 9**<br><br>**40.** Process evaluations should assess the quality of the strategies in place to monitor adherence to protocol (e.g. via a variety of both qualitative and quantitative data recording methods).<br>**Disagree** **Agree**<br>**1 2 3 4 5 6 7 8 9**<br><br>**41.** Participants' understanding of the intervention should be assessed.<br>**Disagree** **Agree**<br>**1 2 3 4 5 6 7 8 9**	Understanding if the intervention has been carried out as initially planned can therefore prove both, very challenging and highly dependent on the quality and level of detailed information included in the plan to execute procedures and assessment. Strategies in order to address this need to be clearly described and in place throughout the research trial.

42. There should be strategies in place to monitor participants' utilisation of the intervention provided.

Disagree **Agree**

1 2 3 4 5 6 7 8 9

43. The process evaluation should collect data regarding participants' experiences of the intervention, and the level of acceptability that was achieved.

Disagree **Agree**

1 2 3 4 5 6 7 8 9

Understanding and interpreting process evaluation results

44. There should be a detailed description of the synthesis of process evaluation findings with trial results.

Disagree **Agree**

1 2 3 4 5 6 7 8 9

45. Theoretical frameworks should be used in order to build explanations that link process and outcome evaluations.

Disagree **Agree**

1 2 3 4 5 6 7 8 9

46. Process evaluations should provide evidence surrounding the chances of Type III errors (implementation failure) at the time of analysing trial's results.

Disagree **Agree**

1 2 3 4 5 6 7 8 9

47. Plans to develop a theory as part of the process evaluation research results should be clearly described

Disagree **Agree**

1 2 3 4 5 6 7 8 9

SUPPORTING INFORMATION:

In regards to the contributions that process evaluations should bring to rehabilitation theory development: process evaluation should provide a clear description of what did or did not work, why it did or did not work and in what way. As a results, it can help to understand and improve theory-informed interventions.

Process evaluation in rehabilitation research can help rehabilitation theory development in two ways:

1. Process evaluation can help understand and critique the theoretical frameworks that were considered at the time of developing the intervention. Process evaluations can therefore contribute to further development and modifications of published frameworks in order to tailor them and make them applicable to rehabilitation research.
2. Process evaluation data and research can also be used to develop new frameworks on how rehabilitation interventions work.

Methodology

48. The design of the process evaluation should be reported in detail.

Disagree **Agree**

1 2 3 4 5 6 7 8 9

49. Ethics and other approvals for process evaluations data collection should be included in the trial approval process.

Disagree **Agree**

1 2 3 4 5 6 7 8 9

50. A process evaluation should use a clear set of measures and evaluation criteria that will need to be described and reasoning behind them provided.

Disagree **Agree**

1 2 3 4 5 6 7 8 9

51. Methods used to investigate the different components of the process evaluation should be reported.

Disagree **Agree**

1 2 3 4 5 6 7 8 9

SUPPORTING INFORMATION:

When rehabilitation researchers decide to carry out a process evaluation they should provide clear details describing how the process evaluation data collection and design are going to be 'linked' to the research trial in order to explain its results.

For example: when embedding process evaluation at the start and throughout a rehabilitation research trial is not possible or feasible (in cases due to budget or staff limitations) the research team might decide that the process evaluation will be carried out as a retrospective analysis or that it will remain at 'arm's length'. This needs to be clearly stated and the rationale behind this decision should be described.

52. Reasoning behind timing for data collection should be clearly stated.

Disagree **Agree**

1 2 3 4 5 6 7 8 9

53. Process evaluation data should be collected from all intervention and control sites.

Disagree **Agree**

1 2 3 4 5 6 7 8 9

54. Process evaluations should use a variety of methods and strategies to gather data, including both qualitative and quantitative approaches.

Disagree **Agree**

1 2 3 4 5 6 7 8 9

55. Details regarding the triangulation of the data within the process evaluation should be clearly reported.

Disagree **Agree**

1 2 3 4 5 6 7 8 9

**56.** Process evaluation protocols should be clearly described and made available.<br>**Disagree** **Agree**<br>**1 2 3 4 5 6 7 8 9**<br><br>**57.** Process evaluation results should be published alongside trial results.<br>**Disagree** **Agree**<br>**1 2 3 4 5 6 7 8 9**	
