# Supplementary material for: The generation of consensus guidelines for carrying out process evaluations in rehabilitation research
Source: BMC Med Res Methodol. 2018 Dec 29;18:180. doi: 10.1186/s12874-018-0647-y (PMC6311071; doi:10.1186/s12874-018-0647-y)
Supplement: Supplementary file 2 — Interview schedule. (PDF 459 kb) [file 12874_2018_647_MOESM2_ESM.pdf]

PRIFYSGOL
BANGOR
UNIVERSITY

CONSENSUS WORK to generate a best practice guidance for carrying out process evaluations within neurological rehabilitation research

Name of Researcher: Patricia Masterson Algar (PhD student)

Phase II – Interview schedule

Thank you very much for taking the time to help with this interview, your feedback as an 'expert in the area' is very valuable to this work.

1. Have you had the time to read the documents that I emailed you regarding this consensus work (Recommendations and systematic review)?
2. Overall, what do you think about the recommendations in general?
    a. Did you think the language used was clear? Why?
    b. Are you familiar with the terminology? Are you familiar with the term 'process evaluation'? What do you understand by 'process evaluation'? Which terms are less clear?
    c. What did you think about the 'flow' and the structure of the document? Do you think the order in which the statements are written makes sense? What changes in the order would you make?
3. Talking about the recommendations in more detail
    a. What do you think about the introduction section stating the 'stand point'?
    b. What do you think about the first section – theoretical approaches?
    c. What do you think about the statements refereeing to the role of process evaluations in understanding the impact of contextual factors?
    d. What do you understand by 'tailoring'? How do you think it should be monitored?
4. Who do you think would benefit from using these guidelines? Would you use these guidelines when carrying out your research? Why?
